# Oscillating glucose induces microRNA-185 and impairs an efficient antioxidant response in human endothelial cells

**DOI:** 10.1186/s12933-016-0390-9

**Published:** 2016-04-30

**Authors:** Lucia La Sala, Monica Cattaneo, Valeria De Nigris, Gemma Pujadas, Roberto Testa, Anna R. Bonfigli, Stefano Genovese, Antonio Ceriello

**Affiliations:** Department of Cardiovascular Research, IRCCS MultiMedica, Milan, MI Italy; Insititut d’Investigacions Biomèdiques August Pi i Sunyer (IDIBAPS) and Centro de Investigación Biomedica en Red de Diabetes y Enfermedades Metabólicas Asociadas (CIBERDEM), Barcelona, Spain; Experimental Models in Clinical Pathology, INRCA-IRCCS National Institute, Ancona, Italy; Scientific Direction, INRCA, Via S. Margherita, 5, Ancona, 60124 Italy

**Keywords:** Oscillating glucose, miR-185, Antioxidant defense, GPx-1, Oxidative stress

## Abstract

**Background:**

Intracellular antioxidant response to high glucose is mediated by Cu/Mn-superoxide dismutases (SOD-1/SOD-2), catalase (CAT) and glutathione peroxidases (GPx), particularly glutathione peroxidase-1 (GPx-1). Although oscillating glucose can induce a more deleterious effect than high glucose on endothelial cells, the mechanism by which oscillating glucose exerts its dangerous effects is incompletely understood; however, the involvement of oxidative damage has been generally accepted. In this study we sought to determine whether oscillating glucose differentially modulates antioxidant response, and to elucidate the potential regulatory mechanisms exerted by the microRNA-185 (miR-185).

**Methods:**

Human endothelial cells were exposed for 1 week to constant and oscillating high glucose. SOD-1, SOD-2, CAT and GPx-1, as well as two markers of oxidative stress [8-hydroxy-2′-deoxyguanosine (8-OHdG) and the phosphorylated form of H2AX (γ-H2AX)] were measured at the end of the experiment. Intracellular miR-185 was measured and loss-of function assays were performed in HUVEC. Bioinformatic tool was used to predict the link between miR-185 on 3′UTR of GPx-1 gene. Luciferase assay was performed to confirm the binding on HUVEC.

**Results:**

After exposure to constant high glucose SOD-1 and GPx-1 increased, while in oscillating glucose SOD-1 increased and GPx-1 did not. SOD-2 and CAT remained unchanged under both conditions. A critical involvement of oscillating glucose-induced miR-185 in the dysregulation of endogenous GPx-1 was found. Computational analyses predict GPx-1 as miR-185′s target. HUVEC cultures were used to confirm glucose’s causal role on the expression of miR-185, its target mRNA and protein and finally the activation of antioxidant response. In vitro luciferase assays confirmed computational predictions targeting of miR-185 on 3′-UTR of GPx-1 mRNA. Knockdown of miR-185, using anti-miR-185 inhibitor, was accompanied by a significant upregulation of GPx-1 in oscillating glucose. 8-OHdG and γ-H2AX increased more in oscillating glucose than in constant high glucose.

**Conclusions:**

Glucose oscillations may exert more deleterious effects on the endothelium than high glucose, likely due to an impaired response of GPx-1, coupled by the upregulation of miR-185.

**Electronic supplementary material:**

The online version of this article (doi:10.1186/s12933-016-0390-9) contains supplementary material, which is available to authorized users.

## Background

It has recently been suggested that glucose variability may be an independent risk factor for vascular complications of diabetes [1, 2]. Recently, glucose oscillations, like those experienced daily by diabetic patients, have been demonstrated to be more dangerous in vitro than constant high glucose; this is true for several cell types, including endothelial cells [3–7]. A higher generation of free radicals during glucose oscillations has been hypothesized as a causal factor for this phenomenon [3–7].

It has been shown that endogenous antioxidant enzymes protect cells against the toxic effect of reactive oxygen species (ROS) and are an essential defence system against oxidative injury [8]. Under normal physiological conditions, ROS production is balanced by an efficient system of antioxidants: such molecules are capable of neutralizing them and thereby preventing oxidant damage. Superoxide anion, a highly reactive molecule, can be converted into less reactive hydrogen peroxide (H_2_O_2_) by the cytosolic Cu/Zn-superoxide dismutase (SOD-1), and by the mitochondrial located Mn-superoxide dismutase (SOD-2), whereas glutathione peroxidase-1 (GPx-1) and catalase (CAT) play a role in the further enzymatic catabolism of ROS [8].

The aim of this study was to compare the response in human endothelial cells of this antioxidant system during glucose oscillation to that which takes place during chronic stable glucose exposure.

A new class of small non-coding RNAs, termed microRNAs (miRNAs or miRs), is emerging as new regulators of metabolism during development and disease [9]. miRNAs are endogenous ~23 nt long, non-coding RNA molecules that play important gene regulatory roles in cells by pairing to the un-translated region (3′-UTR) of mRNAs of protein-coding genes in order to direct their post-transcriptional repression [10]. miRNA recognises complementary miRNA recognition elements (MRE) throughout mRNA sequences, including 3′- and 5′-UTRs. The dysregulation of miRNA expression can affect the expression of hundreds mRNAs and proteins. miRNAs are found in different genomic regions: introns of protein-coding genes; exons and introns of non-coding genes and even the 3′-untranslated region (3′-UTR) of protein-coding genes [12].

Multiple studies have demonstrated that a large number of miRNAs is under the control of various metabolic stimuli, including glucose [11]. Although many miRNAs have already been identified, their roles in the regulation of key genes and signaling pathways associated with glucose stimuli still remain poorly understood. Recently, microRNA-185 (miR-185) has been related to altered expression of selenoproteins, including altered GPx-1 [13], but its direct binding did have not yet been reported. Therefore, in this work, we sought to evaluate the possible modulation carried out by miR-185 during glucose oscillations on GPx-1 expression.

## Methods

### Materials and cell cultures

Primary pooled human umbilical vein endothelial cells (HUVECs) and growth factors were purchased from Lonza (Lonza Bioresearch LBS, Basel, Switzerland). Cells were maintained for 3 weeks in endothelial basal medium, supplemented with low fetal bovine serum (2 %), hydrocortisone (1 µg/mL), basal fibroblastic growth factor (5 ng/mL), epidermal growth factor (5 ng/mL), heparin (0.75 units/mL) and gentamicin/amphotericin (GA-1000, 0.1 %) in a humidified incubator with 5 % carbon dioxide added.

### Experimental design and glucose exposures

2 × 10^5^ HUVECs/well were seeded in 6-well plates (Corning, NY, USA) and exposed for 7 days to three different glucose concentrations: normal glucose (NG; 5 mmol/l), oscillating glucose (OG; 5/25 mmol/l) and high glucose (HG; 25 mmol/l). OG condition was obtained changing glucose concentration (from 5 to 25 mmol/l) every day. Experimental control was performed incubating the cells with mannitol at the same concentration of glucose.

### Determination of 8-hydroxy-2′-deoxyguanosine (8-OHdG)

8-OH-dG, a marker of oxidative stress, was determined in the HUVECs using Bioxytech 8-OHdG-EIA Kit (OXIS Health Products, Portland, OR, USA).

### RNA extraction and Real-time PCR (q-PCR) analysis

Total RNA was extracted using an RNA purification kit (NorgenBiotek, Thorold, ON, Canada). One microgram of total RNA was reverse transcribed using the SuperScript III reverse transcriptase and random hexamers (Invitrogen, Life Technologies, Grand Island, NY, USA). q-PCR was performed using the ABI 7900 HT thermo-cycler (Applied Biosystems, Life Technologies, Grand Island, NY, USA), in a reaction buffer using Taqman Gene expression Master Mix, with pre-optimized primers and probes obtained from Applied Biosystems, and using SYBR green ready mix (SYBR^®^ Premix Ex Taq™ II, Taqara, Japan). The list of primers used is reported in Table 1. All *q*-*PCR* were normalized to actin-beta, as a housekeeping gene.

**Table 1:** List of primers

Gene	Sequence	Accession number
GPx-1	5′-CCCAGTCGGTGTATGCCTTC-3′ 5′-AGCATGAAGTTGGGCTCGAA-3′	NM_000581.2
SOD-2	5′-GGCCTACGTGAACAACCTGA-3′ 5′-CAGGACGTTATCTTGCTGGG-3′	NM_001024465
SOD-1	Hs00533490_m1	NM_000454.4
CAT	Hs00156308_m1	NM_001752.3
ACTB	Hs99999903_m1	NM_001101.3

### Endogenous expression, mimic and inhibition of miR-185

miR-185 expression was examined with the TaqMan MicroRNA Assay Kit (Applied Biosystems, Life Technologies, Grand Island, NY, USA). MultiScribe Reverse Transcriptase was used for RT-PCR, and TaqMan primers for hsa-miR-185 (assay ID 002271) were used to monitor miR-185 expression. RNU44/48 or SNORD-44/48 (assay ID 001094/ID 001006) was used as endogenous miRNA controls (all purchased from Applied Biosystems). has-miR-185-5p *mir*Vana^®^ miRNA mimic (MC12486), Anti-miR™miRNA-185 inhibitor (AM12486), an antisense miR-185, and scrambled Anti-miR™miRNA inhibitor negative control (AM17010) was purchased from Ambion (Foster City, CA, USA). Transfections of miRNA-185 inhibitors were performed at least three times in triplicate using INTERFERin^®^ transfection reagent according to the manufacturer’s protocol (POLYPLUS-transfection, NY, USA).

### Target predictions of miRNAs

The target gene predictions of human miRNAs have been gathered from a publicly available database for miRNAs target predictions (TargetScan 5.2, http://www.targetscan.org, for poorly conserved sites [14]). Sequence for miRNA was obtained from the miRNA database, miRBase (Faculty of Life Sciences, University of Manchester). RNA hybrid tool [15] was used to predict the resulting secondary structure formed by interacting mRNA and miRNA and calculate ΔG minimum free energy. RNA hybrid is available at https://bibiserv.techfak.unibiekefeld.de/rnahybrid.

### HUVEC co-transfection for functional assay

HUVEC 5 × 10^4^ cells passage 4 (p4) were transiently co-transfected with 3′-UTR-GPx-1 expression vector firefly luciferase reporter assay (Origene, MD, USA), containing the entire 217 bp GPx-1 3′-UTR together with miRNA-185 mimic sequence, using jetPRIME co-transfection reagent following manufacturer’s instructions (POLYPLUS). As controls, cells were transfected with empty vector (pMIR) alone, and with pMIR with miRNA-185 mimic sequence. Cells were processed for lysis and collected 72 h after co-transfection and luciferase activity of total cell lysates measured using an established luciferase reporter assay kit (Dual Luciferase Reporter System, Promega, USA). Luciferase values were normalized calculating RLU/μg protein.

### Western immunoblots

Whole cell lysates were prepared using RIPA buffer (Sigma-Aldrich, St. Louis, MO, USA) containing a protease and phosphatase inhibitor cocktail. Protein contents were determined using the Bradford Reagent (Sigma-Aldrich, St. Louis, MO, USA). Whole cell lysates and chromatin-bound nuclear extracts were subjected to 4–20 % Tris–glycine gradient (SDS-PAGE) gels (Lonza Bioresearch LBS, Basel, Switzerland) in reducing conditions and blotted onto a polyvilynidene fluoride membrane. After blocking with 5 % non-fat dry milk in 20 mM Tris–HCl (pH 7.5), 135 mM NaCl, and 0.1 % Tween-20, blots were incubated with polyclonal antibodies against phospho-histone2AX (γ-H2AX) obtained from Cell Signaling (Beverly, MA, USA), washed with 20 mM Tris–HCl (pH 7.5), 135 mM NaCl, and 0.1 % Tween-20, and incubated with a horseradish peroxidase-conjugated secondary antibody. Proteins were detected using the ECL system (Pierce Chemical, Rockford, IL, USA), according to the manufacturer’s instructions and revealed using a CCD camera (ImageQuantLAS4000, GE Healthcare, UK). Antibodies against SOD-1, CAT, GPx-1 (Cell Signaling, Beverly, MA, USA), and SOD-2 (Santa Cruz Biotechnology, CA, USA) were blotted in the same conditions. β-Actin (Sigma-Aldrich, St. Louis, MO, USA) was used as loaded control, whereas histone-H3 (Abcam) was used for a chromatin-bound nuclear extract loaded control. Protein content quantification was performed using computer-assisted densitometry (http://www.imagej.nih.gov, ImageJ, NIH, Bethesda, MD, USA).

### Subcellular protein extracts

Chromatin-bound nuclear extracts were prepared using Subcellular Protein Fractionation Kit for Cultured Cells (Pierce Chemical, Rockford, IL, USA) according to the manufacturer’s description. Thus, HUVECs (2 × 10^6^) cells were fractionated, equal amounts of chromatin-bound nuclear extracts (30 µg) were separated in 4–20 % Tris–glycine gradient gels (Lonza Bioresearch LBS, Basel, Switzerland), and then run on SDS-PAGE in reducing conditions.

### Measurement of GPx activity

HUVECs (1 × 10^6^) were subjected to lysis and sonication (2 × 30 s) on ice. Supernatants were collected and assayed for protein content using Bradford assay. GPx protein activity was measured by GPx activity colorimetric assay kit (Biovision, Milpitas, CA, USA) according to the manufacturer’s instructions and normalized by protein content.

### Statistical analysis

Results are expressed as mean ± SEM. Statistical analysis was performed using GraphPrism5.0^®^ (http://www.graphpad.com). Differences between groups were carried out by one-way ANOVA, followed by the Tukey’s post hoc test. Significant differences were assumed at p < 0.05. Three different experiments were performed in triplicate to ensure reproducibility.

## Results

### GPx-1 response is altered in oscillating glucose

**Fig. 1:**
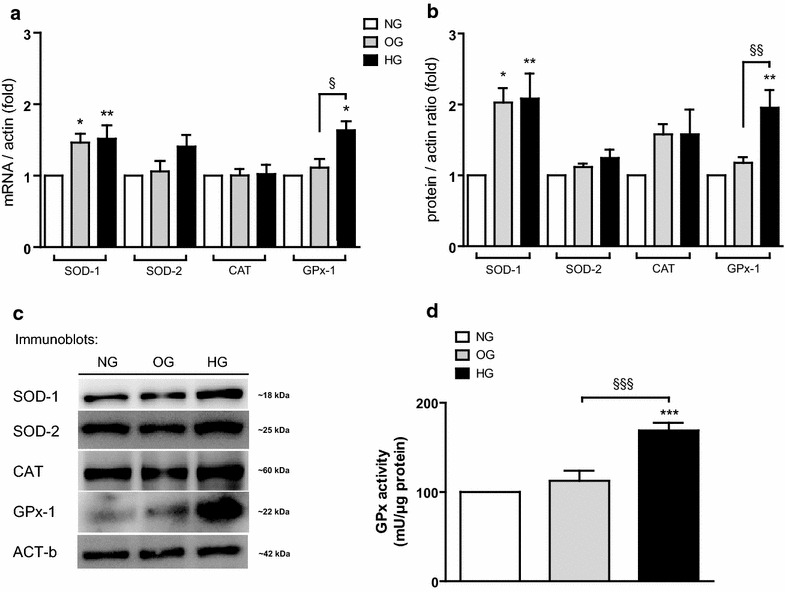
Impaired antioxidant response profiles in HUVECs during high and oscillating glucose exposure. SOD-1, SOD-2, CAT and GPx-1 in OG, HG and NG: **a** mRNA q-PCR real time, **b** densitometry analyses **c** one representative western blot, **d** activity of GPx. Data are expressed as mean (±SEM). *p < 0.05, **p < 0.01, ***p < 0.001 vs control. *Symbols over the bars* refer to differences between the conditions shown *under the bars* (^§^p < 0.05, ^§§^p < 0.01, ^§§§^p < 0.001)

The endogenous expression of SOD-1, SOD-2, CAT and GPx-1 were assessed with *q*-PCR and western blot analysis (Fig. 1a–c). In HG in respect to NG, SOD-1 [mRNA (p < 0.01) and protein *(*p < 0.01)] and GPx-1 [mRNA (p < 0.05) and protein (p < 0.01)] were increased (Fig. 1a–c). In OG in respect to NG, SOD-1 [mRNA (p < 0.05) and protein (p < 0.05)] was increased, while GPx-1 (mRNA and protein) did not change (Fig. 1a–c). This profile of GPx-1 was also confirmed for protein activity (Fig. 1d). After exposure to HG, GPx activity was significantly higher vs NG and OG (p < 0.001). GPx activity was not different in OG vs NG (p = ns). SOD-2 and CAT were unchanged in both conditions, even though there was a tendency for CAT to increase (p = 0.09), (Fig. 1a–c).

### Effects of OG and HG on oxidative stress markers

**Fig. 2:**
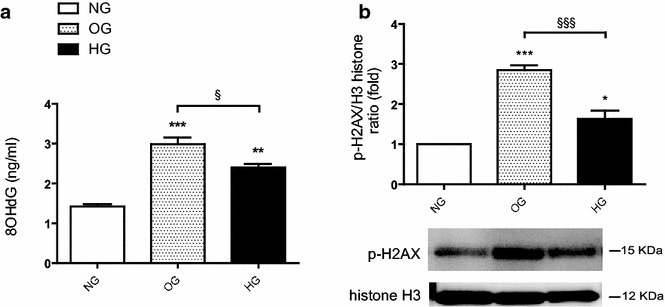
Effects of oscillating and high glucose on oxidative stress markers in HUVECs. **a** 8-OHdG content (ng/ml) in HUVECs during OG, HG and NG. **b** γ-H2AX in HUVECs during OG, HG and NG. The results are expressed as mean (± SEM), (*p < 0.05, **p < 0.01, ***p < 0.001 vs control). *Symbols over the bars* refer to differences between the conditions shown *under the bars* (^§^p < 0.05, ^§§§^p < 0.001)

The 8-OHdG content and γ-H2AX levels in HUVECs, as markers of oxidative damage to nucleic acids, were investigated. Our results shown increased 8-OHdG in both OG (p < 0.001) and HG (p < 0.01) compared to NG (Fig. 2a). However, OG induced more damage on DNA (8-OHdG) than HG (OG vs HG, p < 0.05). Similarly, HG (p < 0.05) and OG (p < 0.001) promoted increased double strand breaks in the cells, demonstrated by γ-H2AX formation (Fig. 2b). OG induced more γ-H2AX than HG (OG vs HG, p < 0.001).

### miR-185 expression is upregulated by OG

**Fig. 3:**
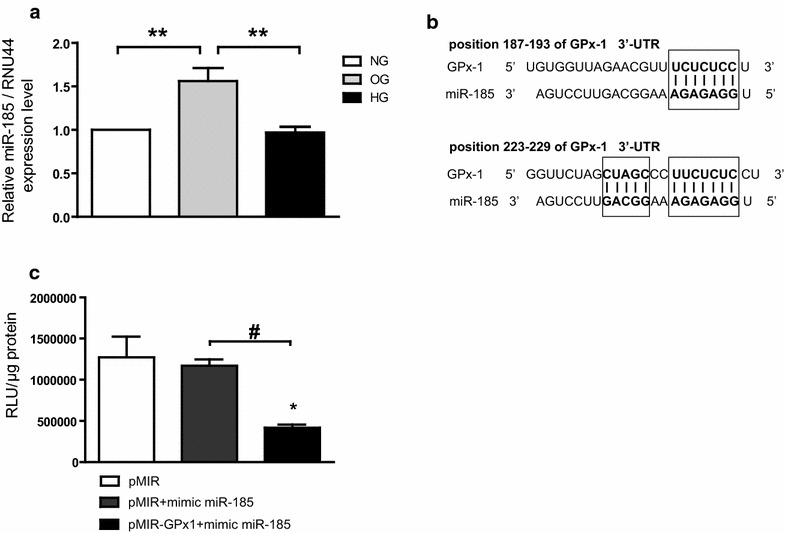
Oscillating glucose-induced miR-185 modulates cellular antioxidant response to oxidative stress. **a** Mean expression values of intracellular miR-185 in OG, HG and NG. **p < 0.01. **b** Sequence alignment between miR-185 and its 2 putative binding sites in the 3′-UTR of GPx-1 mRNA from humans, with sequences recognized by miR-185 seed sequence shown in *bold*. **c** Functional binding assay. The binding of miRNA to the 3′UTR results in repression of luciferase gene expression. Firefly luciferase values were normalized to protein content. Data are expressed as relative light units (RLU) and in relation to empty vector. Mean values (±SEM) of three independent experiments are shown. *p < 0.05 vs pMIR,#p < 0.05 pMIR-GPx1 plus mimic vs pMIR plus mimic

It was first addressed whether miR-185 expression was regulated in response to different glucose levels. In OG, miR-185 expression significantly increased vs NG (p < 0.001) and HG (p < 0.01), while it was unchanged in NG and HG (Fig. 3a).

### GPx-1′s target prediction

The short length of the seed region predicts multiple potential target genes in the genome. Acting as the specificity components of ribonucleoprotein silencing complexes, miRNAs pair with target mRNAs at sites complementary to the miRNA 5′ region. Most effective sites map to 3′ untranslated regions (3′ UTRs) and pair perfectly with the miRNA seed (nucleotides 2–7), with an additional pair at nucleotide 8 and/or an A across from nucleotide 1. Using in silico analysis (TargetScanHuman v.5) to predict the target gene of miR-185, we identified two binding sites into 3′-UTR of the GPx-1 gene (Fig. 3b). Although the target prediction was based on poorly conserved site analysis, the mature miR-185 stranded with the seed region in a perfect alignment between nucleotides 2–7 (Fig. 3b). This region is a key predictor of direct binding to the target gene, as suggested by Lewis BP et al. [16]. The ability of an miRNA to translationally repress a target mRNA is largely dictated by the free energy of binding of the first eight nucleotides in the 5′ region of the miRNA. RNA hybrid analysis of GPx-1 3′-UTR offered further support for direct binding of miR-185 to the site; the predicted binding of miR-185 on GPx-1 3′-UTR had highly favorable predicted minimum free energy scores of ΔG (Gibbs free energy) = −23.4 kcal/mol, consistent with known miRNA targeting [17].

### miR-185 interacts with GPx-1 3′-UTR

In the present study we confirmed the Targetscan in silico predictions demonstrating for the first time a specific and significant interaction between GPx-1 3′UTR and miR-185 using luciferase assay. Co-transfection of HUVEC with GPx-1 3′UTR and miR-185 resulted in a significant down regulation of the luciferase light emission (RLU, Relative Light Unit) compared to cells co-transfected with pMIR alone (p < 0.05), and with pMIR plus mimic miR-185 (p < 0.05) (Fig. 3c). Three independent experiments and two replicated transfections were performed.

### Endogenous GPX-1 levels are differentially modulated in OG rather than HG by miR-185

**Fig. 4:**
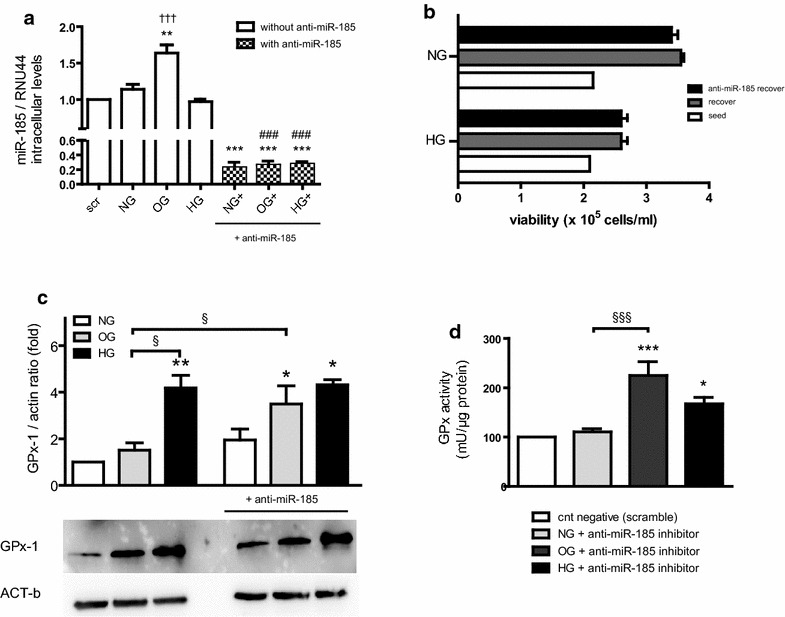
Knock-down of miR-185 under high and oscillating glucose. **a** q-PCR of miR-185 in HUVECs cultured in OG, HG and NG, following transfection with inhibitor of miR-185. Data are means (± SEM, **p < 0.01, ***p < 0.001 vs control;^###^p < 0.001 vs OG vs OG+, and HG vs HG+; ^†††^p < 0.001 OG vs HG on the *left bars*). **b** Number of viable cells in cell suspensions with anti-miR-185 inhibitor. **c** Western blot analysis of GPx-1 with or without anti-miR-185 during NG, OG and HG. **d** GPx activity in HUVECs during miR-185 inhibition in scrambled sample during NG, OG and HG. The results are expressed as mean (±SEM), (*p < 0.05, ***p < 0.001 vs control). *Symbols over the bars* refer to differences between the conditions shown *under the bars* (^§§§^p < 0.01)

Because GPx-1 expression, with respect to NG, was unchanged in OG and increased in HG, we explored the effects of miR-185 on GPx-1 target gene. OG-induced upregulation of miR-185 was associated with unchanged levels of GPx-1, as detected by q-PCR and Western blot (Fig. 1a–c). To define the effects of miR-185 on GPx-1 gene regulation, miR-185 expression was silenced using the miR-185 Anti-miR™ miRNA inhibitor. We transfected 1 nmol/l of anti-miR-185 in HUVECs at the end of culture in NG, OG and HG (Fig. 4a). The transfection efficiencies, assessed by measuring miR-185 expression in q-PCR (Fig. 4a), were ≥80 %. No difference in NG compared with scrambled miRNA transfection was found. Our results demonstrated that the intracellular levels of miR-185 in transfected samples (Fig. 4a) were reduced in a significant manner (OG vs NG, p < 0.01; OG vs HG, p < 0.001; Fig. 4a). Trypan blue exclusion dye also demonstrated no cellular loss during transfections (Fig. 4b; Additional file 1:Figure S1). In anti-miR-185 inhibition transfection to knockdown miR-185, we noticed a significant inverted tendency in GPx-1 protein levels (Fig. 4c), suggesting that GPx-1 was modulated specifically by OG and could be a real target for miR-185. We also measured GPx activity during inhibition of miR-185 and found an increased activity in OG in respect to HG (p < 0.05, Fig. 4d).

## Discussion

In vitro studies report that OG is more dangerous than HG for several kinds of cells [3–7]. Moreover, these studies consistently prove that this is due to an increase in oxidative stress generation in OG compared to HG [3–7].

The intracellular antioxidant defense system plays a key role in protecting cells during the generation of oxidative stress: generally under these conditions there is an increase in key intracellular antioxidant enzymes, such as SODs, CAT and GPx, aiming to protect cells [8]. This response is also active in oxidative stress generated by HG with the only exception of SOD-2, which does not increase [18].

In this study, for the first time, we report that during OG the response of an antioxidant enzyme, GPx-1, is defective compared to what happens in HG. The absence of an increase of GPx-1 in OG, compared to HG, in our opinion, can convincingly explain the more damaging effects of OG on cells as compared to HG. When GPx-1 does not increase, clearly the oxidative stress produced in cells by glucose is more dangerous to the cells. This hypothesis is further supported by our data regarding the markers of oxidative stress, which are more elevated in OG than in HG.

miRNAs are known as important regulators for target mRNA stability and translation. Recently, it has been shown their influence on many cellular functions including glucose metabolism [19]. Important roles for these miRNAs have emerged in the control of metabolic pathways, as suggested by studies implicating miRNAs in the regulation of fat metabolism, adipocyte differentiation, energy homeostasis, and glucose-stimulated insulin secretion [20]. Many studies have identified specific miRNAs expression profiles of diabetes and described the critical roles of miRNAs in insulin secretion [21, 22], pancreatic development and function [22] and diabetic cardiovascular complications [23]. However, the role of several miRNAs, such as miR-21, -146a [24, 25] and let7A [26] as regulators in inflammation and oxidative stresses has been reported.

We have found that OG increases miR-185 expression, a phenomenon that convincingly leads to a decreased expression of its target GPx-1. MIR185 cytogenetic location was found by genomic sequence analysis; Wang et al. (2013) [27] mapped the gene within the first intron of the C22ORF25 gene (also known as Transport and golgi organization 2 homolog, *Tango2*) on chromosome 22q11.21 in sense orientation. Deletion of this region it has been associated with Di George syndrome, and consequently loss of miR-185 contributes to the cardiac defects in the syndrome [28]. In mouse was detected highest relative miR-185 expression in liver and has been related to lipid metabolism [27]. Recent experimentally validated targets for miR-185, such as Camk2d, Ncx1, and Nfatc3 have been related to cardiac diseases [29, 30]. Moreover, IL-10Rα was found a direct target of miR-185, demonstrating a further role in inflammation [31].

Our study suggests a key role of miR-185 in the dangerous effects of OG. If confirmed in vivo, the evidence of a defective GPx-1 response to OG could help explain the mounting evidence linking glucose variability with diabetic complications [1, 2]. In recent years it has emerged the hypothesis that glucose variability can contribute to the development of complications in diabetes. Recently, high blood glucose variability has been defined as an independent determinant of increased lipid and decreased fibrous contents with larger coronary plaque burden [32] and may impact the formation of lipid-rich plaques and thinning of fibrous cap in CAD patients on lipid-lowering therapy [33]. Moreover, one-year visit-to-visit glucose variability predicted development of end stage renal disease in T2DM patients [34]  and was independently associated with the presence of cardiovascular autonomic neuropathy in patients with inadequately controlled T2DM [35]. In our previous study we found that in oscillating and high glucose, total endoglin, its soluble form (sENG), KLF-6 and HIF-1 α were significantly increased [36], and glucose variability reduction via continuous subcutaneous insulin infusion in T1DM increases circulating EPCs levels, suggesting a novel mechanism of vascular damage by oscillating glucose [37].

Consistently, the activity of the antioxidant enzymes CAT, SODs and of GPx has been described as defective in diabetics with complications [38, 39], and an association in vivo has been found between reduced GPx activity and increased risk of cardiovascular complications in diabetes [40].

Glutathione plays a central role in antioxidant defense [41]. Reduced glutathione detoxifies ROS, such as H_2_O_2_, and lipid peroxides, directly or in a GPx-catalyzed mechanism [42]. GPx-1 is an abundant cytoplasmic enzyme specifically involved in the response to peroxyl-radicals and plays an important role in intracellular detoxification. It has been found to be more effective than CAT in removing intracellular peroxides under many different physiological conditions [42, 43]. Moreover, GPx-1 catalyzes the conversion of H_2_O_2_ or organic hydro-peroxides into water, or its corresponding alcohols, using glutathione as a substrate [44], and protects against oxidative and nitrosative stress in blood vessels [45]. H_2_O_2_ forms the toxic oxygen species hydroxyl radical, which is highly reactive and causes lipid peroxidation, and hydroxide anion, which promotes alkaline tissue damage, a process that is offset in part by CAT and GPx-1-dependent reduction to H_2_O. A deficiency in GPx-1 would then lead to an increase in ROS. Thus, a defective GPx-1 response in OG can expose the vasculature to potentially important damage.

## Conclusion

Our study shows for the first time that the exposure of endothelial cells to OG produces an impaired antioxidant response, in particular, that the upregulation of miR-185 contributes to GPx-1 inappropriate response in OG. This finding, in our opinion, contributes to explaining why OG is more dangerous that HG, as reported by several studies [3–7], and further supports the hypothesis that OG may be involved in the development of diabetic complications [1, 2].

## Additional file

10.1186/s12933-016-0390-9 The cells viability after 5 days of exposures to glucose.

## Abbreviations

8-OHdG: 8-hydroxy-2′-deoxyguanosine
CAT: catalase
EBM-2: endothelial basal medium
GPx: glutathione peroxidases
GPx-1: glutathione peroxidases-1
γ-H2AX: phosphorylated form of H2AX
H_2_O_2_: hydrogen peroxide
HG: high glucose
HUVECs: human umbilical vein endothelial cells
microRNAs: miRNAs
miR-185: microRNA-185
MRE: microRNA recognition element
NG: normal glucose
OG: oscillating glucose
ROS: reactive oxygen species
RLU: relative light unit
SOD-1: copper zinc SOD
SOD-2: manganese SOD
3′ UTRs: un-translated regions
