# Supplementary figures and images for: Oscillating glucose induces microRNA-185 and impairs an efficient antioxidant response in human endothelial cells
Source: Cardiovasc Diabetol. 2016 Apr 30;15:71. doi: 10.1186/s12933-016-0390-9 (PMC4852407; doi:10.1186/s12933-016-0390-9)

## Supplemental 1 (S1)

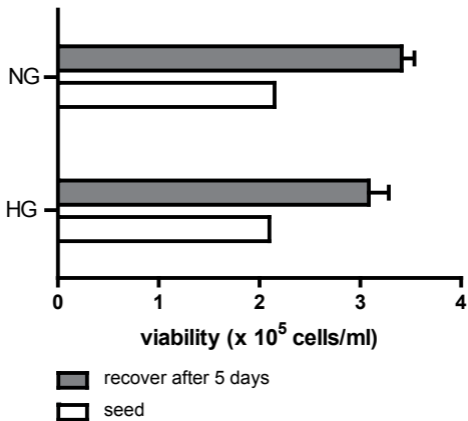

S1. The cells viability after 5 days of exposures to glucose.

Supplement: Supplementary file 1 — 10.1186/s12933-016-0390-9 The cells viability after 5 days of exposures to glucose. [file 12933_2016_390_MOESM1_ESM.pdf]
